# Association of sICAM-1 and MCP-1 with coronary artery calcification in families enriched for coronary heart disease or hypertension: the NHLBI Family Heart Study

**DOI:** 10.1186/1471-2261-7-30

**Published:** 2007-10-26

**Authors:** Weihong Tang, James S Pankow, J Jeffrey Carr, Russell P Tracy, Suzette J Bielinski, Kari E North, Paul N Hopkins, Aldi T Kraja, Donna K Arnett

**Affiliations:** 1 Division of Epidemiology and Community Health, School of Public Health, University of Minnesota, Minneapolis, MN, USA; 2 Department of Radiology, Wake Forest University School of Medicine, Winston-Salem, NC, USA; 3 Department of Pathology and Biochemistry, University of Vermont, Burlington, VT, USA; 4 Department of Epidemiology, University of North Carolina-Chapel Hill, Chapel Hill, NC, USA; 5 Cardiovascular Genetics, University of Utah School of Medicine, Salt Lake City, UT, USA; 6 Division of Statistical Genomics, Washington University School of Medicine, Saint Louis, MO, USA; 7 Department of Epidemiology, School of Public Health, University of Alabama-Birmingham, Birmingham, AL, USA

## Abstract

**Background:**

Data accumulated from mouse studies and in vitro studies of human arteries support the notion that soluble intercellular adhesion molecule-1 (sICAM-1) and monocyte chemoattractant protein-1 (MCP-1) play important roles in the inflammation process involved in atherosclerosis. However, at the population level, the utility of sICAM-1 and MCP-1 as biomarkers for subclinical atherosclerosis is less clear. In the follow-up exam of the NHLBI Family Heart Study, we evaluated whether plasma levels of sICAM-1 and MCP-1 were associated with coronary artery calcification (CAC), a measure of the burden of coronary atherosclerosis.

**Methods:**

CAC was measured using the Agatston score with multidetector computed tomography. Information on CAC and MCP-1 was obtained in 2246 whites and 470 African Americans (mean age 55 years) without a history of coronary heart disease (CHD). Information on sICAM-1 was obtained for white participants only.

**Results:**

In whites, after adjustment for age and gender, the odds ratios (ORs) of CAC (CAC > 0) associated with the second, third, fourth, and fifth quintiles of sICAM-1 compared to the first quintile were 1.22 (95% confidence interval [CI]: 0.91–1.63), 1.15 (0.84–1.58), 1.49 (1.09–2.05), and 1.72 (1.26–2.36) (p = 0.0005 for trend test), respectively. The corresponding ORs for the second to fifth quintiles of MCP-1 were 1.26 (0.92–1.73), 0.99 (0.73–1.34), 1.42 (1.03–1.96), and 2.00 (1.43–2.79) (p < 0.0001 for trend test), respectively. In multivariable analysis that additionally adjusted for other CHD risk factors, the association of CAC with sICAM-1 and MCP-1 was attenuated and no longer statistically significant. In African Americans, the age and gender-adjusted ORs of CAC associated with the second and third tertiles of MCP-1 compared to the first tertile were 1.16 (0.64–2.08) and 1.25 (0.70–2.23) (p = 0.44 for trend test), respectively. This result did not change materially after additional adjustment for other CHD risk factors. Test of race interaction showed that the magnitude of association between MCP-1 and CAC did not differ significantly between African Americans and whites. Similar results were obtained when CAC ≥ 10 was analyzed as an outcome for both MCP-1 and sICAM-1.

**Conclusion:**

This study suggests that sICAM-1 and MCP-1 are biomarkers of coronary atherosclerotic burden and their association with CAC was mainly driven by established CHD risk factors.

## Background

Response of the endothelium to injuries to the arterial wall, including increased expression of inflammatory markers, is a well-recognized component of atherosclerosis [1]. Critical steps involved in the early phase of atherosclerosis include recruitment of leukocytes from the circulation, their attachment to the vascular surfaces and transendothelial migration [2,3]. Monocyte chemoattractant protein-1 (MCP-1) is a chemokine that plays an important role in recruiting circulating monocytes to sites of atherosclerotic lesions [4]. It is produced by a variety of cells including endothelial cells, smooth muscle cells, and macrophages [4]. Intercellular adhesion molecule-1 (ICAM-1), which belongs to the immunoglobulin superfamily, is constitutively expressed on vascular endothelial cells and leukocytes and can be induced on vascular smooth muscle cells [5,6]. ICAM-1, present on the vascular endothelium, participates in leukocyte adhesion and facilitates leukocyte transendothelial migration [2,7]. Soluble ICAM-1 (sICAM-1) is a circulating form of ICAM-1 and has been shown to reflect cell surface expression of ICAM-1 in human vascular endothelial cells [8]. The main mechanism responsible for release of sICAM-1 is believed to be proteolytic cleavage of ICAM-1 from cell membranes [9].

Data accumulated from in vitro and mouse studies support the notion that sICAM-1 and MCP-1 play important roles in atherogenesis. In human coronary atherosclerotic plaques, ICAM-1 is strongly expressed by endothelial cells and macrophages [10]. An examination of human arteries has revealed detectable MCP-1 levels in multiple cell types in atherosclerotic plaques but little expression in normal vessels [11]. Studies in low-density lipoprotein (LDL) receptor- or APOE-knockout mice have shown that a deficiency of ICAM-1 or MCP-1 reduced atherosclerosis [12-14].

Prospective epidemiological studies have found increased risk of incident or recurrent coronary heart disease (CHD) associated with higher baseline levels of sICAM-1 and MCP-1 [15-18], but it is unclear whether sICAM-1 and MCP-1 reflect the burden of subclinical coronary atherosclerosis. Coronary artery calcification (CAC) measured by computed tomography (CT) is a marker of the burden of coronary atherosclerosis and is closely correlated with the extent of total coronary atheroma as measured histologically [19]. There have been limited epidemiologic studies examining the relation between CAC, MCP-1, and sICAM-1 [20-22]. Specifically, the association of CAC with MCP-1 was investigated in only one population-based study [20], and with sICAM-1 in two small studies of selected individuals but with somewhat inconsistent results [21,22]. Moreover, the study populations included in those studies were either predominantly white populations or populations of mixed ethnicity and there were no race-specific data or tests of race interaction in this regard. In this study, we investigated whether CAC was associated with sICAM-1 and MCP-1 in a large population-based biracial sample free of clinical CHD.

## Methods

### Study population

The NHLBI Family Heart Study (FHS) is a population-based family study investigating the genetic and non-genetic determinants of CHD, subclinical atherosclerosis, and cardiovascular disease risk factors [23]. About half of the families in the study were ascertained randomly and the other half selected for a positive family history of CHD. Both samples were recruited through probands in the Framingham Heart Study (Framingham, MA), the Utah Health Family Tree Study (Salt Lake City, UT), and the Atherosclerosis Risk in Communities (ARIC) Study (Minneapolis, MN and Forsyth County, NC). Probands, their spouses, parents, siblings, and children who were over 25 years old were invited to participate.

In its follow-up examination conducted between 2002 and 2003, the FHS recruited about 1/3 of the original families to participate in a second examination to measure calcification of the coronary arteries by cardiac multidetector CT. These families were the largest FHS families and previously genotyped for anonymous genetic markers spanning across the genome. In addition, a new center was established at the University of Alabama-Birmingham to recruit African-American probands from the Hypertension Genetic Epidemiology Network Study and their first-degree relatives [24]. All of the data reported in the study were obtained in the follow-up examination. This study was approved by institutional review board at each participating field center and the coordinating center. Written informed consent was obtained from each participant.

### Study procedures

A clinical examination was performed according to a standardized protocol. Participants were asked to fast for twelve hours prior to their arrival at the study center where their blood was drawn for laboratory tests. During the clinic visit, a standardized interview was conducted to obtain information on medical history and personal behaviors, and anthropometric measurements were made with the participant wearing a scrub suit or examination gown. Blood pressure was measured after participants had rested seated for 5 minutes and taken as the average of the second and third of three measurements using a Dinamap device. Participants were asked to bring all medications taken in the previous 4 weeks and the interviewer transcribed this information. Ever smoking was defined as having smoked at least 100 cigarettes in lifetime. Subjects were categorized into never smokers, former smokers and current smokers. Never smokers were subjects who answered 'no' to the ever-smoking question. Former smokers were subjects who were ever smokers and had quit smoking at the time of the interview. Pack-years of smoking were calculated as the number of packs of cigarettes smoked per day multiplied by the number of years the participant smoked. Use of hormone replacement therapy was defined as having taken or used pills, skin patches, or shots for hormone replacement therapy in the past month.

Measurements of fasting plasma triglycerides, HDL, and total cholesterol were obtained using standard laboratory methods. LDL cholesterol was calculated using the Friedewald formula for subjects with triglycerides concentrations ≤ 400 mg/dL. For subjects with higher levels of triglycerides (n = 52), LDL cholesterol was calculated by an equation developed in 123 FHS participants of the 1^st ^examination who were free of prevalent CHD, had triglycerides > 400 and had LDL cholesterol measured on EDTA plasma by ultracentrifugation LDL cholesterol = 55.7+(-0.358*age)+(4.232*gender)+(0.532*total cholesterol)+(-0.160*HDL cholesterol)+ [-0.614*(triglycerides/5)], where gender is coded as men = 1 and women = 2]. This equation explained 74% of variation in LDL cholesterol and performed better than the Friedewald formula according to correlation coefficient between measured and predicted LDL cholesterol (0.86 vs 0.84), median difference (-0.55 vs -5.0) and maximum difference (-42/70 vs -75/95). Hypertension was defined as systolic blood pressure (SBP) ≥ 140 mmHg, diastolic blood pressure (DBP) ≥ 90 mmHg, or treatment for hypertension. Diabetes was defined by the American Diabetes Association criteria (fasting glucose ≥ 126 mg/dL or use of hypoglycemic medication). CHD was defined by self-reported history of myocardial infarction, coronary angioplasty, or coronary bypass surgery.

### Measurement of calcified atherosclerotic plaque in the coronary arteries

Cardiac CT examinations were performed using the protocol and quality control procedures employed in the Multi-Ethnic Study of Atherosclerosis (MESA) and Coronary Artery Risk Development in Young Adults (CARDIA) [25]. In brief, biweekly calibration scans documenting the stability of each CT system's calibration to water and 3 concentrations of calcium were obtained to monitor accuracy, precision, and temporal drift. On-site training and certification were performed. Technical parameters were: 120 KV, 106 mAs, 2.5 mm slice collimation, prospective ECG gating at 50% of the R-R interval, 0.5 or 0.8 second gantry rotation, a partial scan reconstruction resulting in a temporal resolution of between 250–500 msec and a 35 cm display field-of-view. All participants were imaged with a calcium calibration standard within the imaging field (Image Analysis, Columbia, KY). For participants weighing 100 kg (220 lbs) or greater, the milliampere setting was increased by 25%. The scan through the heart was performed twice sequentially with a 1-minute interval. The effective radiation exposure for the average participant was 1.5 mSv for men and 1.9 mSv for women.

Calcified atherosclerotic plaque in the epicardial coronary arteries was analyzed centrally at Wake Forest University Health Sciences, Winston Salem, by trained image analysts using dedicated hardware and software covering the entire heart (GE Healthcare Advantage Windows Workstation with SmartScore, Waukesha, WI). Analysts measured calcified plaque in the epicardial coronary arteries with the program calculating a total calcium or Agatston score modified to account for slice thickness using a 130 CT number threshold and a minimum lesion size of 0.9 mm (i.e., 2 pixel connectivity filter). The total calcium scores from the first and second CT scan series were then averaged, and the average score was used in the analysis.

### Measurement of inflammation biomarkers

Inflammation biomarkers were measured at the Department of Pathology and Biochemistry, University of Vermont. MCP-1 was measured in citrated plasma using an ultra-sensitive ELISA assay (Quantikine Human MCP-1 Immunoassay; R&D Systems, Minneapolis, MN). Inter-batch coefficients of variation (CVs) ranged from 4.6 – 6.7%. sICAM-1 was measured in citrated plasma by an ELISA assay (Parameter Human sICAM-1 Immunoassay; R&D Systems, Minneapolis, MN). The laboratory CV was 5.0%. C-reactive protein (CRP) was measured in SCAT-I plasma by a BNII nephelometer (Dade Behring, Deerfield, IL) utilizing a particle enhanced immunonepholometric assay. Inter-batch CVs ranged from 2.1% – 5.7%.

### Statistical analysis

There were 3111 subjects who had complete information on CAC score, sICAM-1 and MCP-1. We excluded 395 subjects who met the following criteria: 1) other race groups than white or African American (n = 18); 2) African Americans from the North Carolina field center or whites from the Birmingham center (n = 13); 3) prevalent CHD or history of coronary revascularization procedures at the time of the study (n = 351); 4) outliers with log-transformed sICAM-1 or MCP-1 values greater than or less than 5 standard deviations from the corresponding mean (n = 13). Thus, 2716 subjects (2246 whites and 470 African Americans) were included in the analyses. We excluded subjects with prevalent CHD or history of coronary revascularization procedures because the focus of the study is on subclinical coronary atherosclerosis. In addition, subjects with prevalent CHD may have undergone treatment that could affect the variables of interest.

It was reported that a K29M mutation at the ICAM-1 gene causes low detectability for serum sICAM-1 level as measured by R&D systems assay which uses the BBE1B antibody [26]. This mutation occurred at a frequency of 20% in African Americans and 0.4% in Caucasians [26]. Therefore, the 470 African-American participants were excluded from the analysis of sICAM-1. Accordingly, the study population for MCP-1 comprised both whites and African Americans and that for sICAM-1 comprised whites only.

Comparison of distributions of demographic and clinical characteristics between those with and without CAC was made by generalized estimating equations (GEE), which account for familial relatedness. Spearman rank order correlations were used to assess the association of sICAM-1 and MCP-1 with continuous covariates. The association of categorical covariates with sICAM-1 and MCP-1 was evaluated in GEE in which natural log-transformation was performed for sICAM-1 and MCP-1 to normalize the trait distributions. Accordingly, sICAM-1 and MCP-1 are presented as geometric means with 95% confidence intervals. All of the above and subsequent analyses for MCP-1 were stratified by race. To investigate the association between the inflammation markers and CAC, we estimated the odds ratio (OR) of presence of CAC across sICAM-1 and MCP-1 categories by GEE models and computed p values for trend using a continuous variable that was assigned the median value of sICAM-1 or MCP-1 in each exposure category. In whites, sICAM-1 and MCP-1 were grouped into quintile levels, while tertile categories were used for MCP-1 in African Americans due to smaller sample size. Furthermore, we applied tertile cutpoints of MCP-1 in African Americans to whites and evaluated whether the ORs of CAC associated with each level of MCP-1 differed between African Americans and whites. Confounding was assessed in 3 sequential models: no adjustment; partial adjustment: adjusted for age and gender; multivariable adjustment: adjusted for age, gender, field center, BMI, SBP, diabetes, HDL and LDL cholesterol, triglycerides, physical activity index (met-min/week), hours of television watched/day, smoking status (current, former, never), pack-years of smoking, current alcohol consumption, and hormone replacement therapy. We initially tested the interaction of sex on the association between inflammation markers and CAC in the above GEE analyses, and did not observe significant interaction. Therefore, the above analyses were conducted with men and women combined. In addition, we tested whether the association of CAC with sICAM-1 and MCP-1 differed across age groups (tertile groups in whites and median groups in African Americans) and did not observe significant age differences in either race group.

Since presence of CAC according to Agatston score > 0 may be subject to measurement errors due to tissue-associated artifacts, we chose two different criteria in the above analyses for defining the presence of CAC (CAC score > 0 and CAC score ≥ 10). The cut point of CAC score ≥ 10 was chosen to be consistent with other studies addressing similar research questions [20].

Finally, among whites subjects with detectable CAC (CAC > 0), a linear regression model implemented in GEE was used to test for trend with log-transformed CAC score as the dependent variable and sICAM-1 and MCP-1 as independent variables. The natural log-transformed CAC score was approximately normally distributed. This analysis was not conducted for MCP-1 in African Americans due to limited number of African Americans with CAC > 0. All analyses were performed using SAS version 8.2 (SAS Institute).

## Results

In whites, the mean ± SD, median and inter-quartile range for sICAM-1 (ng/ml) are 246.7 ± 78.6, 231.3, and 195.1~281.6, respectively, and for MCP-1 (pg/ml) were 189.1 ± 60.5, 180.2, and 149.9~218.9, respectively. In African Americans, the mean ± SD, median and inter-quartile range for MCP-1 (pg/ml) were 174.3 ± 68.3, 168.2, and 136.5~195.0, respectively. The mean level of MCP-1 was significantly lower in African Americans than in whites, and this difference persisted after adjusting for age, gender, BMI, SBP, diabetes, HDL and LDL cholesterol, triglycerides, physical activity index (met-min/week), hours of television watched/day, smoking status (current, former, never), pack-years of smoking, current alcohol consumption, and hormone replacement therapy (p < 0.01). The prevalence of hypertension was 39.3% in whites and 75.1% in African Americans.

Table 1 shows demographic and clinical characteristics of subjects by CAC status. Compared to subjects with zero calcium score, subjects with detectable calcium were older and more likely to be males, current or former smokers, and have hypertension and diabetes. All of the other risk factors, except race, LDL cholesterol, current alcohol consumption, and aspirin use, were also significantly associated with CAC status.

**Table 1:** Demographic and clinical characteristics by CAC status: mean ± SD or percentage

Characteristics	CAC = 0 (N = 1250)	CAC > 0 (N = 1466)	p
Age, years	48.8 ± 10.6	60.4 ± 11.9	<0.0001
Females, %	71.4	51.3	<0.0001
African Americans, %	19.4	15.5	0.33
Body mass index, kg/m^2^	28.5 ± 6.1	30.2 ± 6.2	<0.0001
Waist circumference, cm	94.7 ± 16.1	102.9 ± 15.9	<0.0001
Systolic blood pressure, mm Hg	115.6 ± 18.0	127.0 ± 21.4	<0.0001
Diastolic blood pressure, mm Hg	69.3 ± 10.2	72.3 ± 10.4	<0.0001
Hypertension, %	31.3	57.6	<0.0001
Diabetes, %	6.9	15.4	<0.0001
HDL-C, mg/dL	52.2 ± 14.8	48.9 ± 14.3	<0.0001
Triglycerides*, mg/dL	124.4 ± 84.8	145.6 ± 93.0	<0.0001
LDL cholesterol, mg/dL	112.1 ± 32.7	113.7 ± 33.9	0.18
CRP*, mg/L	3.87 ± 6.02	4.37 ± 6.69	<0.0001
sICAM-1*†, ng/ml	237 ± 68	255 ± 85	<0.0001
MCP-1*, pg/ml	172.1 ± 56.1	198.9 ± 64.3	<0.0001
Pack-years of smoking*‡	14.8 ± 16.8	26.5 ± 25.1	<0.0001
Smoking status			<0.0001
Current smoker, %	9.9	13.2	
Former smoker, %	22.3	34.3	
Never smoker, %	67.8	52.5	
Current alcohol consumption, %	50.9	51.0	0.29
Hormone replacement therapy§, %	23.4	26.1	0.03
Aspirin use, %	23.3	38.5	0.08
Cholesterol lowing medications, %	8.9	26.4	<0.0001
Hours of TV watched/day	2.40 ± 1.74	2.95 ± 2.06	0.0003
Physical activity index*, met-min/week	755.7 ± 1107.6	657.6 ± 992.9	0.008
CAC (mean)	0	282.4 ± 655.2	-
CAC [median (IQR)]	0.0 (0.0–0.0)	48.5 (3.5–256.5)	-

All statistical comparison was adjusted for age and gender except when age or gender were analyzed as an independent variable; the analysis of gender was adjusted for age only;

*Natural log-transformed values were used in the statistical test;

†White participants only: n = 1007 in CAC = 0 group, and 1239 in CAC > 0 group;

‡Current or former smokers only: n = 402 in CAC = 0 group, and 696 in CAC > 0 group;

§Females only: n = 892 in CAC = 0 group, and 752 in CAC > 0 group.

Table 2 presents partial Spearman correlation coefficients of MCP-1 and sICAM-1 with continuous covariates. Due to smaller sample size, the correlations between MCP-1 and the continuous covariates were generally less significant in African Americans than in whites. However, the pattern of correlations agreed between the two racial groups for most of the covariates except that MCP-1 was less strongly associated with BMI, SBP, and physical activity index in African Americans.

**Table 2:** Adjusted* spearman correlation coefficients of sICAM-1 and MCP-1 with continuous covariates

Covariates	sICAM-1_whites_		MCP-1_whites_		MCP-1_AAs_†	
			
	r	P	r	P	r	P
Age	0.11	<0.0001	0.35	<0.0001	0.35	<0.0001
Body mass index	0.09	0.0015	0.17	<0.0001	0.08	0.09
Waist circumference	0.09	0.0001	0.18	<0.0001	0.11	0.02
Systolic blood pressure	0.07	0.0018	0.11	<0.0001	0.03	0.49
Diastolic blood pressure	0.02	0.26	0.05	0.01	-0.03	0.45
HDL cholesterol	-0.13	<0.0001	-0.19	<0.0001	-0.13	0.004
Triglycerides	0.13	<0.0001	0.22	<0.0001	0.18	0.0001
LDL cholesterol	-0.04	0.07	0.04	0.05	0.06	0.19
Pack-years of smoking	0.11	<0.0001	0.02	0.34	0.03	0.57
Physical activity index	-0.04	0.05	-0.11	<0.0001	-0.05	0.30
Hours of television watched/day	0.04	0.07	0.07	0.001	0.07	0.11
MCP-1	0.13	<0.0001	1.00	-	1.00	-
CRP	0.14	<0.0001	0.09	<0.0001	0.11	0.02

*Adjusted for age and gender; the analysis of age was not adjusted for covariates;

†AAs, African Americans.

Table 3 presents adjusted geometric means of MCP-1 and sICAM-1 by categorical covariates. The pattern of associations between MCP-1 and the categorical covariates were consistent between the two racial groups. It is interesting to note that at each level of the categorical variables, mean MCP-1 was consistently lower in African Americans than in whites, further confirming our earlier observation that African Americans had lower levels of MCP-1 compared to whites.

**Table 3:** Adjusted geometric means* (95% confidence interval) of sICAM-1 and MCP-1 by categorical covariates

	sICAM-1_whites_	MCP-1_whites_	MCP-1_AAs_
Gender†			
Female	241.1 (236.9, 245.5)	175.8 (172.4, 179.2)	164.5 (158.8, 170.4)
Male	234.1 (229.4, 238.8)	186.0 (182.4, 189.7)	167.9 (158.4, 178.0)
P	0.01	<0.0001	0.51
Hypertension			
Yes	244.4 (239.2, 249.7)	186.0 (182.0, 190.2)	168.0 (161.0, 175.3)
No	233.2 (228.9, 237.6)	177.5 (174.2, 180.9)	160.7 (151.6, 170.3)
P	0.0006	0.0004	0.19
Diabetes			
Yes	256.0 (245.4, 267.1)	189.8 (182.5, 197.3)	174.6 (162.9, 187.2)
No	235.8 (232.2, 239.5)	180.0 (177.1, 182.9)	163.7 (157.6, 170.0)
P	0.0003	0.009	0.08
Smoking			
Current smoker	282.6 (272.6, 293.0)	201.8 (194.1, 209.8)	171.9 (162.4, 182.0)
Former smoker	234.1 (229.1, 239.1)	175.8 (171.5, 180.1)	157.4 (149.1, 166.3)
Never Smoker	232.0 (227.7, 236.4)	180.0 (176.8, 183.2)	169.1 (161.3, 177.3)
P	<0.0001	<0.0001	0.03
Alcohol Consumption			
Yes	237.5 (232.9, 242.3)	176.9 (173.3, 180.6)	164.0 (156.3, 172.1)
No	237.7 (232.8, 242.6)	185.6 (182.1, 189.2)	168.2 (160.1, 176.7)
P	0.97	0.0002	0.45
Cholesterol-lowering Medication			
Yes	240.0 (234.1, 246.0)	192.6 (187.3, 198.1)	176.6 (159.5, 195.5)
No‡	242.9 (234.7, 249.7)	191.0 (185.5, 196.7)	168.9 (159.2, 179.2)
P	0.64	0.65	0.42
Aspirin Use			
Yes	236.3 (230.9, 241.9)	179.2 (175.3, 183.3)	163.3 (152.0, 175.4)
No	238.2 (233.9, 242.6)	181.6 (178.4, 184.9)	167.0 (160.7, 173.6)
P	0.57	0.29	0.55
Hormone Replacement Therapy§			
Yes	235.1 (228.1, 242.4)	170.9 (165.6, 176.5)	160.2 (149.7, 171.5)
No	243.6 (238.8, 248.4)	179.5 (175.7, 183.3)	167.3 (160.9, 173.9)
P	0.04	0.006	0.28

*Adjusted for age and gender unless otherwise noted below;

† Adjusted for age;

‡Those with total cholesterol ≥ 240 mg/dL and not on cholesterol-lowering medications;

§Females only.

Tables 4 and 5 present ORs of presence of CAC by quintiles of sICAM-1 and MCP-1, respectively, in whites. Based on a criterion of CAC score > 0, coronary calcification was present in 55% (1239) of subjects. When the cut point was raised to CAC ≥ 10, the prevalence of CAC was 37%. There was a significant and positive association between the presence of CAC, sICAM-1 and MCP-1 before and after adjusting for age and gender (Tables 4 and 5). This association was consistent for both the CAC > 0 and CAC ≥ 10 criteria. However, after additionally adjusting for other CHD risk factors, the association between CAC and sICAM-1 was no longer significant whether CAC > 0 or CAC ≥ 10 were analyzed as an outcome (Table 4). In the multivariable adjustment models, the association between CAC and MCP-1 was borderline significant for CAC > 0 and not significant for CAC ≥ 10 (Table 5).

**Table 4:** Odds ratios (95% Confidence Interval) of CAC > 0 or CAC ≥ 10 by sICAM-1 quintile in whites

	Quintiles of SICAM-1					
	
	Q1	Q2	Q3	Q4	Q5	p trend
Subjects	448	450	450	449	449	
Range (ng/ml)	≤ 187.6	187.7 – 217.4	217.5 – 247.1	247.2 – 296.6	> 296.6	
Prevalence of CAC > 0, %	47.3	53.1	53.8	58.8	62.8	
OR of CAC > 0						
No Adjustment	1.00	1.27 (0.99 1.64)	1.29 (0.99 1.68)	1.59 (1.23 2.05)	1.83 (1.40 2.40)	<0.0001
Partial Adjustment*	1.00	1.22 (0.91 1.63)	1.15 (0.84 1.58)	1.49 (1.09 2.05)	1.72 (1.26 2.36)	0.0005
Multivariable Adjustment**	1.00	1.12 (0.82 1.54)	0.98 (0.70 1.36)	1.28 (0.91 1.80)	1.20 (0.86 1.69)	0.21
						
Prevalence of CAC ≥ 10, %	29.5	34.4	38.0	38.8	44.8	
OR of CAC ≥ 10						
No Adjustment	1.00	1.25 (0.94 1.66)	1.46 (1.11 1.92)	1.50 (1.15 1.96)	1.92 (1.47 2.50)	<0.0001
Partial Adjustment*	1.00	1.13 (0.81 1.59)	1.36 (0.97 1.93)	1.31 (0.92 1.88)	1.88 (1.34 2.63)	0.0003
Multivariable Adjustment**	1.00	1.06 (0.75 1.50)	1.19 (0.83 1.68)	1.10 (0.76 1.59)	1.34 (0.94 1.91)	0.13

CAC = coronary artery calcium score;

* Adjusted for age and gender;

** Additionally adjusted for field center, BMI, SBP, diabetes, HDL and LDL cholesterol, triglycerides, physical activity index, hours of TV watched/day, smoking status (current, former, never), pack-years of smoking, alcohol consumption, and hormone replacement therapy.

**Table 5:** Odds ratios (95% confidence interval) of CAC > 0 or CAC ≥ 10 by MCP-1 quintile in whites

	Quintiles of MCP-1					
	
	Q1	Q2	Q3	Q4	Q5	p trend
Subjects	449	449	450	449	449	
Range (pg/ml)	≤ 142.1	142.2 – 167.8	167.9 – 193.7	193.8 – 230.3	> 230.3	
Prevalence of CAC > 0, %	35.4	50.8	52.7	63.9	73.1	
OR of CAC > 0						
No Adjustment	1.00	1.89 (1.45 2.47)	2.03 (1.57 2.62)	3.28 (2.53 4.25)	5.05 (3.81 6.69)	<0.0001
Partial Adjustment*	1.00	1.26 (0.92 1.73)	0.99 (0.73 1.34)	1.42 (1.03 1.96)	2.00 (1.43 2.79)	<0.0001
Multivariable Adjustment**	1.00	1.12 (0.79 1.61)	0.88 (0.63 1.22)	1.12 (0.79 1.59)	1.43 (1.00 2.03)	0.048
						
Prevalence of CAC ≥ 10, %	21.6	31.9	34.4	43.4	54.1	
OR of CAC ≥ 10						
No Adjustment	1.00	1.71 (1.25 2.35)	1.93 (1.44 2.59)	2.84 (2.13 3.78)	4.45 (3.29 6.01)	<0.0001
Partial Adjustment*	1.00	0.99 (0.66 1.47)	0.78 (0.54 1.14)	0.97 (0.66 1.42)	1.44 (0.97 2.14)	0.023
Multivariable Adjustment**	1.00	0.84 (0.54 1.30)	0.73 (0.48 1.11)	0.85 (0.56 1.29)	1.15 (0.76 1.75)	0.19

CAC = coronary artery calcium score;

* Adjusted for age and gender;

** Additionally adjusted for field center, BMI, SBP, diabetes, HDL and LDL cholesterol, triglycerides, physical activity index, hours of TV watched/day, smoking status (current, former, never), pack-years of smoking, alcohol consumption, and hormone replacement therapy.

**Figure 1:**
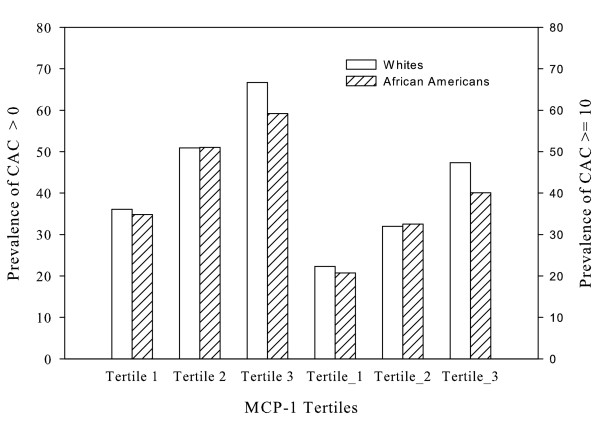
Prevalence of CAC (%) across MCP-1 tertiles by race. The three bar pairs on the left hand side are for CAC > 0 and those on the right hand side are for CAC ≥ 10. The tertile cutpoints for MCP-1 were based on the African-American sample and applied to the white sample.

Table 6 presents ORs of presence of CAC by tertiles of MCP-1 in African Americans, in which the percentage of CAC > 0 and CAC ≥ 10 was 48% and 31%, respectively. Similar to whites, MCP-1 was positively associated with the presence of CAC in unadjusted models. This association was no longer significant after adjustment for age and gender or additional adjustment for other CHD risk factors. Figure 1 shows prevalence of CAC (%) across MCP-1 tertiles in African Americans in comparison to whites, in which MCP-1 tertile cutpoints from African Americans were used to group participants. As shown in Figure 1, the prevalence of CAC > 0 or CAC ≥ 10 increased with MCP-1 in a similar fashion between African Americans and whites; consistent with this observation, tests of interactions did not detect significant interactions of race on the associations between MCP-1 and CAC in raw (no adjustment), partial or multivariate adjustment models (p > 0.05).

**Table 6:** Odds ratios (95% Confidence Interval) of CAC > 0 or CAC ≥ 10 by MCP-1 tertile in African Americans

	Tertiles of MCP-1			
	
	T1	T2	T3	p trend
Subjects	156	157	157	
Range (pg/ml)	≤ 143.6	143.7 – 184.7	>184.7	
Prevalence of CAC > 0, %	34.6	51.0	59.2	
OR of CAC > 0				
No Adjustment	1.00	2.00 (1.21 3.32)	2.76 (1.71 4.48)	<0.0001
Partial Adjustment*	1.00	1.16 (0.64 2.08)	1.25 (0.70 2.23)	0.44
Multivariable Adjustment**	1.00	1.00 (0.53 1.88)	0.82 (0.44 1.54)	0.53
				
Prevalence of CAC ≥ 10, %	20.5	32.5	40.1	
OR of CAC ≥ 10				
No Adjustment	1.00	2.02 (1.13 3.62)	2.78 (1.64 4.69)	0.0001
Partial Adjustment*	1.00	1.03 (0.52 2.04)	1.11 (0.56 2.21)	0.75
Multivariable Adjustment**	1.00	0.86 (0.41 1.82)	0.71 (0.34 1.47)	0.35

CAC = coronary artery calcium score;

* Adjusted for age and gender;

** Additionally adjusted for BMI, SBP, diabetes, HDL and LDL cholesterol, triglycerides, physical activity index, hours of TV watched/day, smoking status (current, former, never), pack-years of smoking, alcohol consumption, and hormone replacement therapy.

Among whites with CAC > 0, there was a positive trend between the amount of CAC and levels of sICAM-1 and MCP-1. However, this association did not reach statistical significance in either partial or multivariable adjustment models (data not shown).

The above results did not change materially after we substituted hypertension for SBP, waist circumference for BMI, and fasting glucose for diabetes in the multivariable adjustment models. Finally, since it is of concern that measurement errors in the estimated LDL cholesterol for those with triglycerides > 400 mg/dL may affect our ability to control for confounding, we replaced LDL cholesterol by total cholesterol in the multivariable analyses and all results were similar (data not shown).

## Discussion

In this large population-based sample free of clinical CHD, we investigated the association of CAC with MCP-1 in both African Americans and whites, and with sICAM-1 in whites. In whites, we found that sICAM-1 and MCP-1 were significantly and positively associated with presence of CAC after controlling for age and gender. However, the observed association was attenuated and no longer statistically significant after additionally adjusting for other CHD risk factors. In African Americans, the magnitude of association between MCP-1 and CAC was similar to that in whites, but less significant due to smaller sample size. While MCP-1 was significantly lower in African Americans than whites independently of other CHD risk factors, the magnitude of associations of MCP-1 with CAC did not differ between the two racial groups.

The association of MCP-1 with coronary calcification has been investigated by Deo et al in a population-based sample of 3,499 adults free of CHD [20]. They found a significant association between MCP-1 and the presence of coronary calcification after adjusting for traditional risk factors that did not include age. This association was not significant after additionally adjusting for age or when age was adjusted for as a sole confounder. In contrast to the Deo et al study, we detected a significant association between CAC and MCP-1 after adjusting for age and gender in whites, although we also observed that the adjustment for age explained most of reduction in the strength of association between CAC and MCP-1 in the partial adjustment model (data not presented). Nevertheless, both studies lead to similar conclusion that MCP-1 is not significantly associated with subclinical atherosclerotic burden after controlling for the influence of established CHD risk factors that include age.

Moreover, both studies consistently observed that MCP-1 was significantly lower in African Americans than in whites. This finding, in conjunction with the previous report of lower extent of CAC in the former group [27], makes it reasonable to hypothesize that the association between MCP-1 and CAC may differ between the two racial groups. In the study by Deo et al, there was no evaluation of interaction effects of race on the association between the two risk variables. Our study extends the Deo et al study by showing that the magnitude of the association between the two risk variables was similar between African Americans and whites.

To our knowledge, our study is the largest to examine the association of CAC with sICAM-1. Two smaller studies have previously examined the association of CAC with sICAM-1 in subjects free of clinical CHD [21,22]. The first was conducted by Folsom et al in 360 subjects who were selected randomly or because of a high carotid intima-media thickness (IMT) from two field centers of the ARIC Study [21]. Coronary calcium score measured in 1999–2000 was analyzed in relation to sICAM-1 measured in 1987–1988. There was no significant association between sICAM-1 and CAC category after adjusting for age, sex and field center. The second study was conducted by Reilly et al in 632 subjects who were recruited because of a family history of premature CHD and a low risk factor profile that excluded those with diabetes, total cholesterol > 300 mg/dL, cigarette smoking > one pack per day, and blood pressure > 160/100 mm Hg [22]. sICAM-1 was significantly and positively associated with CAC score after adjusting for age and gender, but not after further adjustment for other traditional risk factors. While the population included in this study had a different risk factor profile than our study, the results from the two studies are consistent.

Our findings that the association of CAC with sICAM-1 and MCP-1 was independent of age and gender but not independent of other established CHD risk factors suggest that the underlying biological mechanisms that mediate the association between sICAM-1, MCP-1 and coronary calcification are mainly driven by established CHD risk factors. Indeed, many stimuli that promote ICAM-1 and MCP-1 expression are related to CHD risk factors. For example, the expression of ICAM-1 and MCP-1 can be up-regulated by interleukin (IL)-1, IL-6, tumor necrosis factor α, activated platelets, angiotensin II, shear stress, oxidized LDL, and other oxidant species [4,5,7,28,29], all of which have been linked to risk factors for CHD. Moreover, sICAM-1 was reported to be associated with brachial artery reactive hyperemia in the Framingham Offspring Study [30]. Reactive hyperemia is a measure of microvascular vasodilator function and reflects non-endothelium-dependent and possibly endothelium-dependent vascular function [30]. Therefore, findings from our and other similar studies will provide valuable information in the understanding of pathophysiological mechanisms for coronary atherosclerosis.

### Limitations

First, data from our study cannot provide information on the source of circulating ICAM-1 and MCP-1 (i.e., whether it was mainly derived from endothelial cells in the coronary arteries, activated leukocytes or other sources). It is unlikely that these markers were elevated due to major myocardial events because participants in our study were free of clinically apparent CHD. Second, our data are cross-sectional and therefore we cannot determine the temporal sequence between development of CAC and up-regulation of inflammation biomarkers. Data from follow-up studies are needed to determine the utility of these makers in predicting the development of CAC. Third, coronary calcium is a poor marker of coronary stenosis or prognostically significant coronary disease. It has low sensitivity in detecting atherosclerotic lesions that are not calcified and thus it is an imperfect measure of coronary atherosclerotic burden [31]. Fourth, only white subjects were included in the analysis of sICAM-1 and therefore the generalizability of the study findings for sICAM-1 to other ethnicities is uncertain. Finally, the lack of significant race interaction on the association between CAC and MCP-1 needs to be interpreted with caution due to limited sample size for African Americans.

## Conclusion

In a large population-based sample free of clinical CHD, plasma levels of sICAM-1 and MCP-1 were significantly associated with coronary atherosclerosis as measured by CAC. In whites, this association was independent of age and gender, but not of other established CHD risk factors. In African Americans, the magnitude of association between MCP-1 and CAC was similar to that in whites, and there was no significant interaction of race on the association between the two risk variables. Our study suggests that sICAM-1 and MCP-1 are biomarkers of coronary atherosclerotic burden and their association with CAC was mainly driven by established CHD risk factors.

## Competing interests

The author(s) declare that they have no competing interests.

## Authors' contributions

WT, JP, PH, and DA conceived of and designed the study. WT performed the statistical analyses and drafted the manuscript. WT, JP, SB, KN, and AK interpreted the results. JJC performed and read cardiac the CT scans. RT measured the inflammation biomarkers. All authors revised the manuscript critically for intellectual content, and read and approved the final manuscript.

## Pre-publication history

The pre-publication history for this paper can be accessed here:


